# Nervous system–gut microbiota–immune system axis: future directions for preventing tumor

**DOI:** 10.3389/fimmu.2025.1535955

**Published:** 2025-05-01

**Authors:** Juanjuan Wang, Wenyue Cheng, Rongcun Yang

**Affiliations:** ^1^ Department of Immunology, Nankai University School of Medicine, Nankai University, Tianjin, China; ^2^ State Key Laboratory of Medicinal Chemical Biology, Nankai University, Tianjin, China; ^3^ Translational Medicine Institute, Affiliated Tianjin Union Medical Center of Nankai University, Nankai University, Tianjin, China

**Keywords:** nervous system, gut microbiota, immune cells, tumor environment, signal transduction

## Abstract

Tumor is one of the leading causes of death worldwide. The occurrence and development of tumors are related to multiple systems and factors such as the immune system, gut microbiota, and nervous system. The immune system plays a critical role in tumor development. Studies have also found that the gut microbiota can directly or indirectly affect tumorigenesis and tumor development. With increasing attention on the tumor microenvironment in recent years, the nervous system has emerged as a novel regulator of tumor development. Some tumor therapies based on the nervous system have also been tested in clinical trials. However, the nervous system can not only directly interact with tumor cells but also indirectly affect tumor development through the gut microbiota. The nervous system-mediated gut microbiota can regulate tumorigenesis, growth, invasion, and metastasis through the immune system. Here, we mainly explore the potential effects of the nervous system–gut microbiota–immune system axis on tumorigenesis and tumor development. The effects of the nervous system–gut microbiota–immune system axis on tumors involve the nervous system regulating immune cells through the gut microbiota, which can prevent tumor development. Meanwhile, the direct effects of the gut microbiota on tumors and the regulation of the immune system by the nervous system, which can affect tumor development, are also reviewed.

## 1 Introduction

Tumor is one of the leading causes of death worldwide. The occurrence and development of tumors are related to multiple factors such as depression (1), inhibition of the immune system, and alteration of the gut microbiota (2).

There has been a growing interest in the study of depression in patients with tumors, aiming to assess the association between tumors and depression (3). Although a common underlying pathophysiological mechanism between depression and tumors is yet to be elucidated (4), studies have implicated that there exists an altered gut microbiota in anxiety, stress, and depression (5). Evidence from preclinical and clinical studies has suggested that alterations of the gut microbiota play a key role in the pathophysiology of depression (6). Since tumor and depression are closely interrelated, particularly in patients with advanced cancer, who often present with depression (3), an altered gut microbiota in depression should be also related to tumor development.

The effects of gut microbiota on the occurrence and development of tumors have been widely reviewed (2, 7). Multiple factors associated with the gut microbiota such as gut dysbiosis, metabolites, specific pathogenic microbes, and virulence factors can contribute to the initiation and progression of tumors such as colorectal cancers (CRCs). An altered gut microbiota is found in various CRC stages, including early and advanced CRCs. Metagenomic and metabolomic analyses showed distinct phenotypes in gut microbiota in CRCs (8). In CRC patients, *Fusobacterium nucleatum* (*F. nucleatum*) enrichment, short-chain fatty acid (SCFA) depletion, a shift in acetate/acetaldehyde metabolism toward acetyl-CoA production, and reduced microbial GABA (gamma-aminobutyric acid) biosynthesis were noted (8). Furthermore, specific bacteria associated with CRCs were also found. They included *Escherichia coli* (*E. coli*), *F. nucleatum*, *Enterococcus faecalis* (*E. faecalis*), *Streptococcus gallolyticus* (*S. gallolyticus*), and *Bacteroides fragilis* (*B. fragilis*). The metabolites from gut microbiota can not only directly affect tumor development but also play an important role in tumor-associated immune responses such as inflammation and immune tolerance.

The immune system plays a critical role in preventing the occurrence and development of tumors, including immune-effective cells such as CD8 and NK cells; inflammatory cells such as inflammatory macrophages (iMACs), dendritic cells (iDCs), T helper (Th1) cells, and Th17 cells; and immune regulatory cells such as myeloid-derived suppressor cells (MDSCs), immune T regulatory cells (Treg cells), Breg cells, and innate lymphocytes (ILCs). Recent studies involving bio-information analysis also identified some immune cell-associated genes in tumor cells, which could affect tumorigenesis such as DACH1 (9) in benign prostatic hyperplasia; ITGB1 (10), HSP90B1 (11, 12), and pre-mRNA processing factor 19 (PRPF19) (13) in bladder carcinoma; and the monocyte-related gene CCNA2 (14) in prostate adenocarcinoma (PRAD). Importantly, the differentiation and function of these immune cells could be regulated by gut microbiota metabolites (GMMs) such as SCFAs (15–17), tryptophan-derived metabolites (18–20), and bile acid (BA) derivatives (21–24). For example, BAs and their derivatives can affect the differentiation and function of MACs, DCs, MDSCs, Tregs, and T helper (Th) 17 cells (2, 7). Notably, immune cells regulated by the gut microbiota can influence tumor development.

Recent studies have shown that the nervous system, including the central nervous system (CNS) and the peripheral nervous system, plays a role in the progression of various cancers such as gastric, colon, rectal, oral squamous cell, head and neck, prostate, breast, and pancreatic cancers (3), and that the autonomic nerve contributes to prostate cancer progression (25–29). The nervous system can directly affect tumorigenesis, and the nervous system-mediated immune responses also influence the tumor (30, 31). The nervous system–gut microbiota–immune system axis affects tumors where the nervous system regulates immune cells through the gut microbiota to prevent tumor development. This review mainly explores the potential effects of the nervous system–gut microbiota–immune system axis on tumorigenesis and tumor development. Moreover, we also review the direct effects of the gut microbiota on tumors as well as the direct effects of the nervous system on the immune system, which can affect tumor development.

## 2 The enteric nervous system

**Figure 1:**
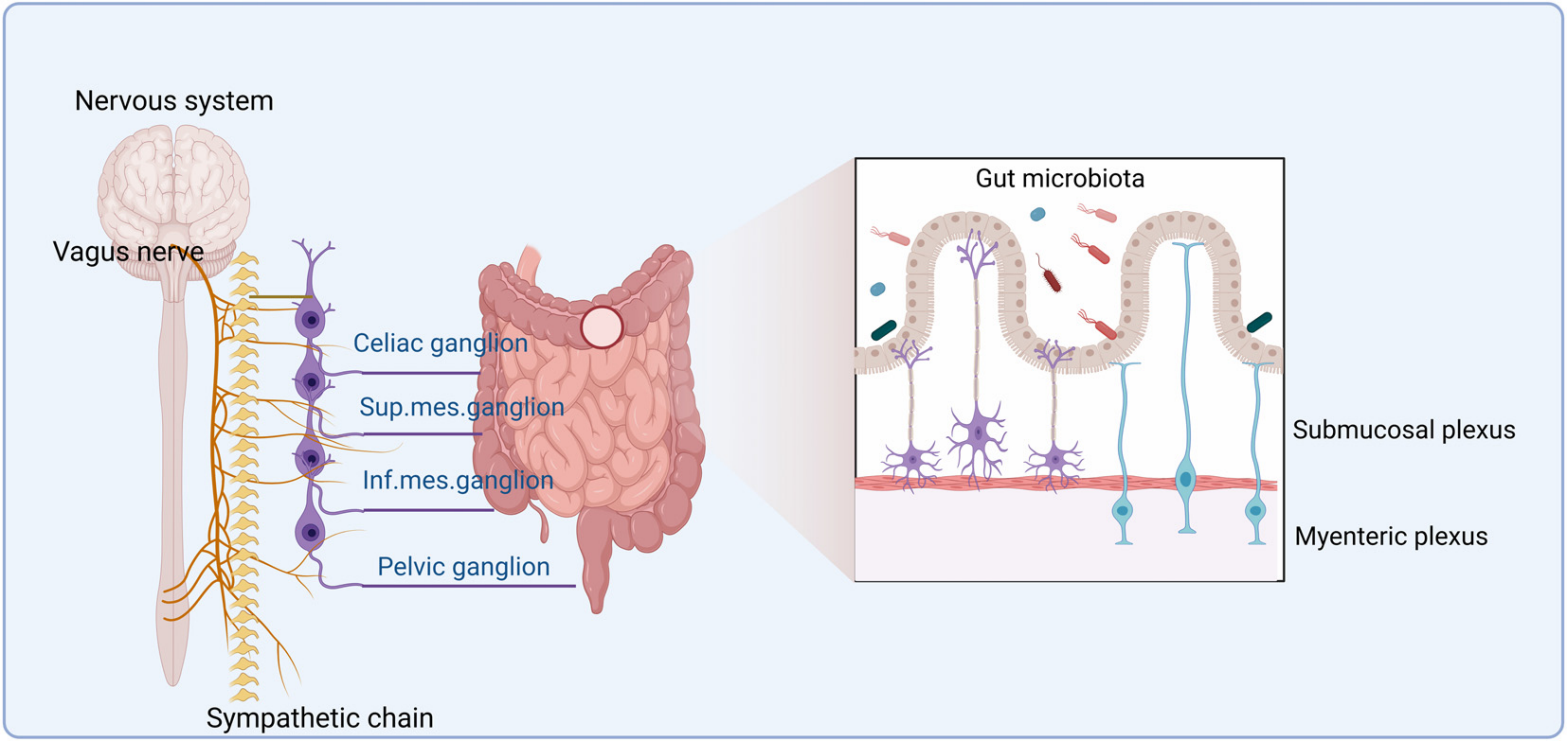
The enteric nervous system. The enteric nervous system (ENS) is formed by the myenteric plexus and submucosal plexus. It receives inputs from both the sympathetic and parasympathetic/vagal nervous systems. The ENS can communicate with the central nervous system (CNS), which encompasses the brain and the spinal cord. After leaving the hindbrain, the parasympathetic vagus nerve travels along the esophagus, through the diaphragm, and ultimately synapses onto the gut tract. On the other hand, the sympathetic nerves originate in the spinal column and synapse onto the sympathetic visceral ganglia, such as the celiac, superior, and inferior mesenteric ganglia. Nervous signals reach the gut microbiota via neurons from the ENS in the submucosa and myenteric plexus of the gut wall.

The enteric nervous system (ENS), a division of the peripheral nervous system (PNS), is in the gut wall. It includes two main plexuses: the myenteric plexus and the submucosal plexus (32) (**Figure 1**). The ENS receives inputs from both the sympathetic and parasympathetic/vagal nervous systems (33), which enter the gastrointestinal (GI) tract (5). Motor neurons in the myenteric plexus mainly control muscle movements, whereas neurons in the submucosal plexus regulate secretion and absorption (34). Both neurons in the myenteric plexus and submucosal plexus can affect the composition of gut microbiota. Notably, recent progress in single-cell transcriptomics has enabled the discovery of new neuronal subtypes and improved the previous cell-type classifications (35, 36).

Although the ENS is capable of functioning autonomously, it also communicates with the CNS, which encompasses the brain and spinal cord (5). The human CNS is the most complex tissue, comprising approximately 86.1 billion neurons in the brain and spinal cord in male individuals, along with a roughly equal number of glial cells (37). CNS canonical activity includes neurotransmitter release at electrochemical synapses and consequent membrane depolarization, which can affect cellular functions (38, 39). Extrinsic connectivity from the CNS to the ENS is dependent on both sympathetic and parasympathetic/vagal nerve fibers (5). After leaving the hindbrain, the parasympathetic vagus nerve can travel along the esophagus onto the GI tract through the diaphragm and ultimately synapses. The sympathetic nerve originates in the spinal column and synapses onto the sympathetic visceral ganglia, such as the celiac, superior, and inferior mesenteric ganglia. Both parasympathetic and sympathetic nerves can directly synapse into the myenteric ganglia (5). Notably, to and from the gut, most neurological signals are through a bidirectional vagus nerve (VN). Signals from the CNS can reach 500 million neurons in the ENS (40) along efferent VN fibers. Bidirectional communication between the ENS and CNS can maintain homeostasis (41). In addition, in the colon, there exist afferent nerves linked to the spinal cord. The pelvic nerves, which are derived from the spinal cord and leave via the sacral spinal nerve, innervate the distal colon and rectum (5).

## 3 Potential effects of the nervous system–gut microbiota–immune system axis on tumors

**Figure 2:**
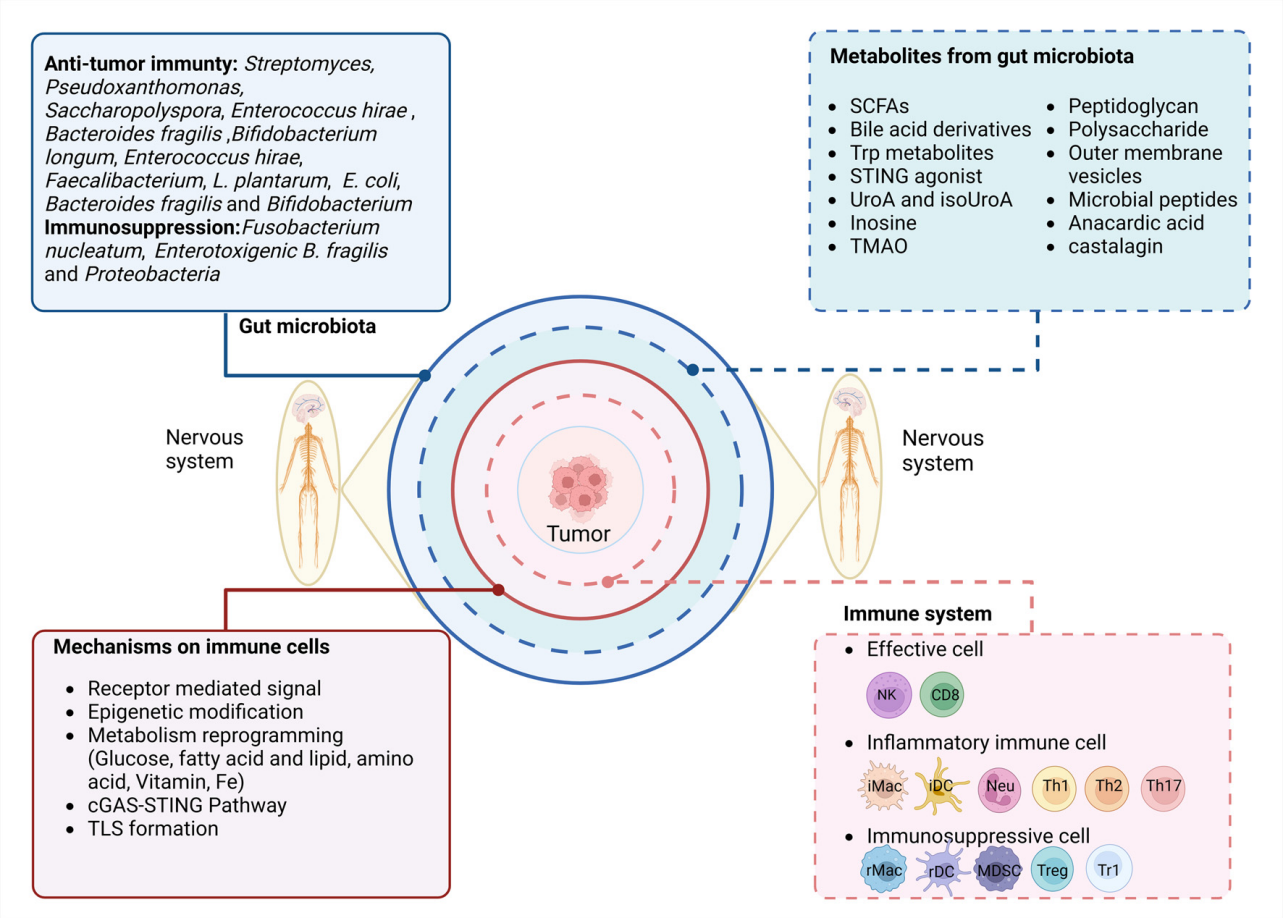
Regulation of the nervous system–gut microbiota–immune axis in tumorigenesis and tumor development. The nervous system–gut microbiota–immune system axis includes the nervous system, the gut microbiota/metabolites, and the immune system. The potential effect of the immune system on the tumor is through nervous system-mediated gut microbiota. They include three parts: 1) effects of the nervous system on gut microbiota/metabolites; 2) regulation of gut microbiota/metabolites in the immune system through multiple pathways such as receptor-mediated signal, epigenetic modification, metabolism reprogramming, cGAS-STING pathway, and tertiary lymphoid structure (TLS) formation; and 3) the effects of the immune system on the tumors.

Some environmental factors can influence the composition of the gut microbiota, including diet, use of drugs, host genetics, and hygiene (42). However, the gut microbiota is also affected by other factors such as the nervous system. The nervous system–gut microbiota–immune system axis includes the nervous system, gut microbiota, and immune system. The effects of the nervous system–gut microbiota–immune system axis on tumors involve the nervous system regulating immune cells through the gut microbiota, which can prevent tumor development, including the effects of the nervous system on gut microbiota, gut microbiota on the immune system, and immune system on tumors (**Figure 2**).

### 3.1 Regulation of the enteric nervous system in gut microbiota

The nervous system can affect the occurrence and development of tumors by regulating the gut microbiota.

#### 3.1.1 CNS and gut microbiota

Accumulating evidence from preclinical and clinical studies has shown that there exists altered gut microbiota in anxiety, depression, and stress (5, 43). The disorders were characterized by lower short-chain fatty acid-producing bacteria (e.g., *Faecalibacterium*) and a higher abundance of pro-inflammatory species (e.g., *Enterobacteriaceae* and *Desulfovibrio*) (43). In depressed patients, microbial metabolites such as butyrate decreased. Furthermore, butyrate could also exert antidepressant actions when administered to rodents, reducing stress responsiveness (44). The level of BAs modulated by the microbiota was inversely correlated with the severity of depression symptoms (45). Other microbial metabolites such as lipopolysaccharide (LPS) and trimethylamine-N-oxide from choline were also associated with depression (46). Neurotransmitters [e.g., serotonin or γ-aminobutyric acid (GABA)] from microbes can also regulate depression (44). The abnormal composition of gut microbiota also appeared in different animal models of depression (47). The restoration of gut microbiota with prebiotics or probiotics has gained considerable attention for the management of depression (48). Notably, dissimilar stressors had different effects on the composition of gut microbiota (49, 50).

#### 3.1.2 ENS and gut microbiota

ENS is essential for the regulation of gut microbiota. Multiple molecular constituents from the ENS can potentially affect the composition of gut microbiota such as acetylcholine, catecholamines, neuropeptides, serotonin and histamine, and neurotrophic factors (5). Since ENS neurons do not extend into the intestinal lumen, they play a role in the microbiota through indirect pathways such as microbial molecules, which can penetrate the epithelial barrier (51). In addition, some microbial molecules also affect the composition of gut microbiota by affecting intestinal motility (52, 53). Notably, the effect of the nervous system on the gut microbiota is largely dependent on specific receptors and neuroregulatory substances in bacteria (54).

Notably, the gut microbiota can communicate with the CNS through specific metabolic compounds, e.g., SCFAs, BA derivatives, GABA, glutamate (Glu), norepinephrine (NE), dopamine (DA), serotonin (5-HT), and histamine. Afferent VN fibers transport signals from the gut microbiota to the brain. In response to these stimuli, the brain sends signals back to enteroepithelial cells via efferent VN fibers. There is no direct contact between VN fibers and intestinal microbiota. The nervous signals are via neurons to reach the gut microbiota from the ENS in the submucosa and myenteric plexus of the gut wall (55). So far, several studies have suggested the effects of ETS on gut microbiota (32), e.g., specific subsets of peripherally activated neurons could regulate the gut microbiome in mice without signal involvement from the brain (56). They found that activating gut ChAT^+^ and TH^+^ neurons of mice could alter the transcriptional and proteomic landscape of the intestines as well as the gut metagenome and metabolome (56). Deletion of choline acetyltransferase in enteric neurons also caused postnatal intestinal dysmotility and dysbiosis (57).

### 3.2 Regulation of the gut microbiota in the immune system

**Figure 3:**
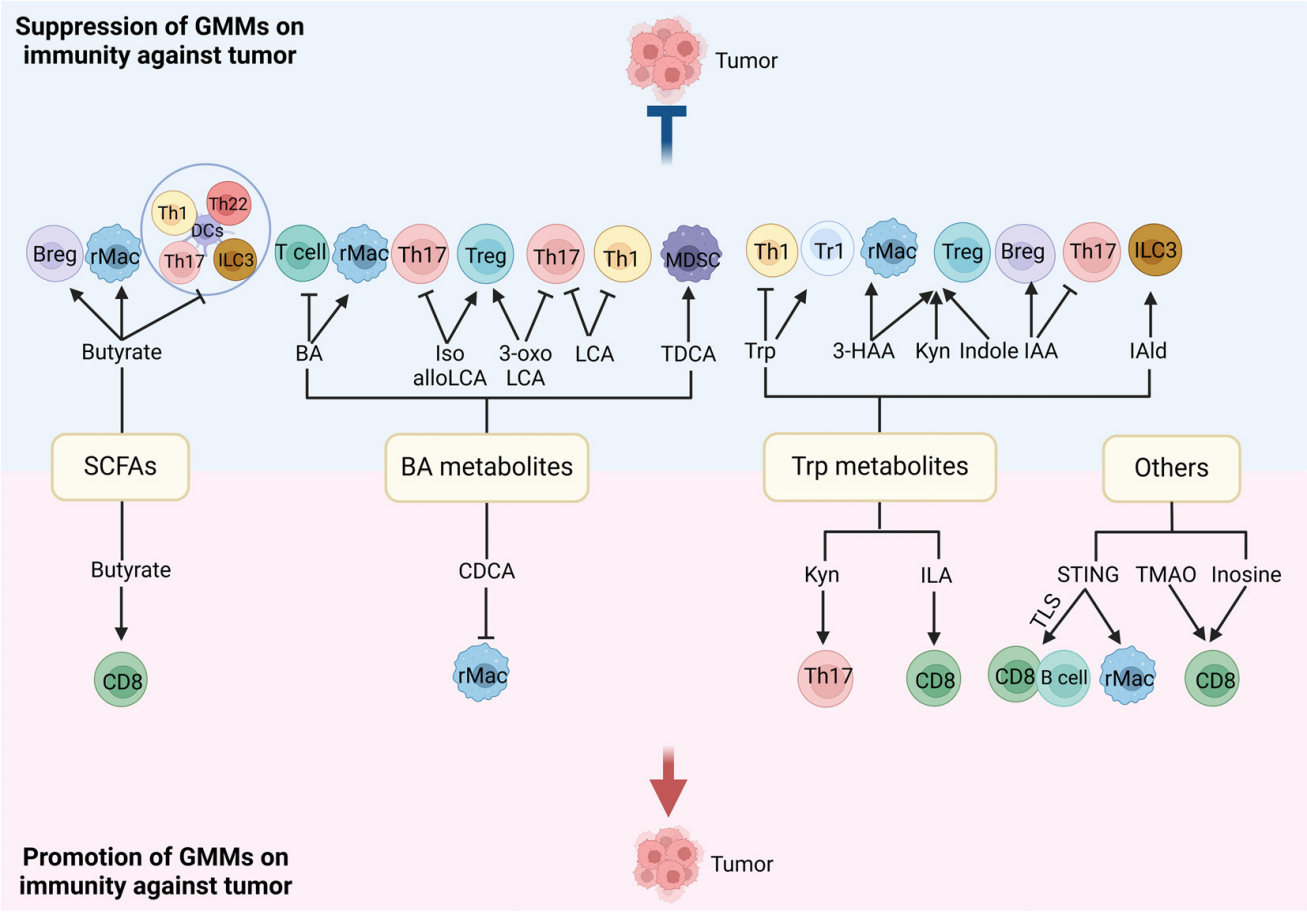
Regulation of gut microbiota metabolites (GMMs) in different immune cells. The gut microbiota/GMMs also have direct negative and positive effects on the function and differentiation of immune cells. Tregs, regulatory T cells; rMACs, regulatory MACs; Bregs, regulatory B cells; MDSCs, myeloid-derived suppressive cells; DCs, dendritic cells; NK cells, natural killer cells; Th cells, T helper cells; ILCs, innate lymphocytes; SCFAs, short-chain fatty acids; CDCA, chenodeoxycholic acid; Trp, tryptophan; Kyn, kynurenine; ILA, indole-3-lactic acid; IPA, indole-3-propionic acid; 3-HAA, 3-hydroxyanthranilic acid; IAA, indole-3-acid-acetic; I3A, indole-3-aldehyde; NorUDCA, norursodeoxycholicacid; STING, stimulator of the interferon gene; TMAO, trimethylamine oxide, TLS, tertiary lymphoid structure.

Gut microbiota plays a critical role in the differentiation and function of immune cells, which can influence tumorigenesis. In germ-free mice, there indeed exist immunological defects such as smaller Peyer’s patches and lymphoid follicles, abnormal germinal center, and reduced protective IgA (58). *Segmented filamentous bacterium* can promote Th17 cell differentiation (59), while *Clostridium* spp. induce Treg cells (60). The commensal bacterial antigens could help shape immunoglobulin repertoires in the gut (61). Thus, microbial signals from both local and distant sources have the ability to regulate innate and adaptive immune responses, causing systemic or tumor local-specific immune regulation (62). They have vast effects on the function and differentiation of immune-effective cells such as effective CD8 and NK cells, immune regulatory (suppressive) cells such as MDSCs, regulatory dendritic cells (rDCs), regulatory macrophages (rMACs), Tregs, Bregs, and also inflammatory cells such as Th1, Th2, and Th17. The regulation of the gut microbiota and its metabolites on immune cells is shown in **Figure 3** (2, 7).

#### 3.2.1 Gut microbiota/its metabolites

The gut microbiome is a fragile ecosystem, composed of bacteria, fungi, protozoans, viruses, and archaea. In many instances, the gut microbiota can regulate tumor progression through antitumor immunity and immunosuppression (63). Studies have also found that *B. fragilis* (64), *B. fragilis* (65) and *Bifidobacterium* (66, 67), *B. fragilis* and *E. coli* (68), *Enterococcus hirae* and *Bifidobacterium longum* (69), *Enterococcus hirae*, *Faecalibacterium* and *Lactiplantibacillus plantarum* (68), *Streptomyces*, *Pseudoxanthomonas*, and *Saccharopolyspora* (70) are related to antitumor immunity, whereas enterotoxigenic *B. fragilis* (71), *F. nucleatum* (72, 73), and *Proteobacteria* (74) were related to immune tolerance and immunosuppression.

Gut microbiota metabolites such as SCFAs, BA derivatives, and tryptophan metabolites can also regulate the differentiation and function of immune cells. SCFAs, mainly including acetate, propionate, and butyrate, are from dietary fiber fermentation in the cecum and colon by gut bacteria (75, 76). BAs are classified into primary BAs such as cholic acid and chenodeoxycholic acid and secondary BAs such as deoxycholic acid (DCA) and lithocholic acid (LCA). Primary BAs are synthesized in the liver. The secondary BAs are formed by bacteria in the colon. Bacteria can also conjugate chenodeoxycholic acid (CDCA), DCA, or cholic acid (CA) to one or more amino acids such as alanine, arginine, and aspartate (77). Conjugated BAs can be deconjugated in gut bacteria (78) and converted into secondary BAs, i.e., DCA and LCA. Four distinct ways, namely, deconjugation, dehydroxylation, oxidation, and epimerization, are used to transform BAs in humans (79). A range of iso-, oxo-, and epi-derivatives (80) such as oxo-BA metabolites 3-oxoLCA, 7-oxoCDCA, 12-oxoCA, 7-oxoCA, 12-oxoDCA (81), iso-LCA, 3-oxo-LCA, 3-oxoallo-LCA, isoalloLCA, allo-LCA, and 3-ketoLCA are also found in gut bacteria (79, 82, 83). Tryptophan metabolites such as indole (84), indole-3-acid-acetic (IAA) (85–87), indole-3-propionic acid (IPA) (85–87), indole-3-propionic acid (IPA) (88), indoleacrylic acid (IA) (88), indole-3-aldehyde (IAld) (89), skatole (87, 90), and tryptamine can be generated by different gut bacteria. In addition, gut microbiota bacteria also generate Kyn and downstream metabolites such as 3-hydroxyanthranilic acid (3-HAA) (91) through encoding enzymes homologous to those of the eukaryotic kynurenine (Kyn) pathway.

STING agents from *Akkermansia muciniphila* and *Lactobacillus rhamnosus* (92, 93), inosine from *A. muciniphila* and *Bifidobacetrium pseudolongum* (94), gut microbiota-derived inosine (94), and sulfur-metabolizing bacteria associated with hydrogen sulfide (81) can improve the activation of immune cells. TMAO from the gut microbiota trimethylamine (TMA) promoted CD8^+^ T-cell-mediated antitumor immunity via pyroptosis in mouse models (95). Other gut microbiota-derived metabolites such as peptidoglycan, polysaccharide, microbial peptides, anacardic acid, outer membrane vesicles, and castalagin could also promote the differentiation and function of inflammatory cells.

Urolithin A may alleviate colitis in mice by triggering AhR activation, improving gut microbiota dysbiosis, and regulating microbial Trp metabolism (96). *Bifidobacterium*-derived urea (97) was also associated with immunosuppression and tolerance to promote tumor development.

#### 3.2.2 Mechanism(s) of the gut microbiota/its metabolites to regulate immune cells

The regulation of gut microbiota and/or its metabolites in immune cells is through receptor-mediated signals, epigenetic modification, metabolism (glucose, fatty acid and lipid metabolism, amino acid, vitamin, and Fe) reprogramming, cGAS-STING (stimulator of interferon genes) pathway, and formation of tertiary lymphoid structures (TLSs).

##### 3.2.2.1 Receptor-mediated signals

SCFAs exert their role in immune cells through their receptors such as the G-protein-coupled receptor (GPR). BA metabolites depend on receptors such as TGR5 (GPBAR1), FXR (farnesol-X-receptor), VDR (vitamin D receptor), LXR (liver X receptor), PXR (pregnane X receptor), and RORγt (retinoid-related orphan receptor γt) to regulate the differentiation and function of immune cells (20, 98), whereas Trp metabolites are through their receptors such as AhR (99).

##### 3.2.2.2 Epigenetic modification

Epigenetic modifications, including post‐translational modifications on histone and non‐histone proteins, can cause downstream cellular responses through regulating gene expression (100, 101). For example, altered gut microbiota in the body have also been shown to participate in the pathogenesis of bowel diseases by regulating the balance of Th17/Tregs through epigenetic modification (102).

##### 3.2.2.3 Metabolism reprogramming

Glycolysis and mitochondrial oxidative phosphorylation (OXPHOS) are basically metabolic pathways for the generation of ATPs. It can rapidly produce energy and supply biosynthetic precursors to facilitate the function of immune cells such as the survival of proliferating cells (103).

Fatty acid and lipid metabolisms are also involved in the differentiation and function of immune cells. For example, the increased biosynthesis of fatty acids was implicated in macrophage polarization by *Bacteroides thetaiotaomicron*-derived acetic acid. Fatty acid oxidation contributes to the production of interleukin (IL)-17 and IL-22 in ILC3s. Elevated fatty acid uptake has been observed in activated ILC3s (104).

Amino acid metabolism also affects immune cells, e.g., glutamine metabolism was required for Tfh cells to perform optimally and interact with GC B cells (105). *Lactobacillus reuteri* with a tryptophan-rich diet could reprogram intraepithelial CD4^+^ T cells into Treg cells (89).

Vitamin D can regulate innate and adaptive immune cells to exert anticancer effects and can influence cancer cell growth, differentiation, and apoptosis through various mechanisms in the tumor microenvironment (TME) (106).

Recent studies have shown that Fe metabolism is also important in the differentiation and function of immune cells. Fe3^+^ enters the cell through the transferrin receptor. It is reduced to Fe2^+^ within the cell, related to ferroptosis. For ferroptosis caused by free iron, lethal lipid peroxides can initiate ferroptosis through catalyzing non-enzymatic Fenton reaction for directly peroxidating polyunsaturated fatty acid-containing phospholipids (107). Ferroptosis, as a new type of cell death, participates in the pathogenesis of diseases. Notably, recent studies have suggested that gut microbiota could regulate ferroptosis in immune cells, e.g., gut microbiota Trp metabolites IDA could inhibit ferroptosis through upregulating ALDH1A3 (aldehyde dehydrogenase 1 family member A3) to generate NADH through AhR. ALDH1A3 is essential for ferroptosis-suppressor protein 1(FSP1)-mediated synthesis of coenzyme Q10 (108).

##### 3.2.2.4 The cGAS-STING pathway

The cGAS-STING pathway can trigger a robust IFN response to enhance therapeutic antitumor immune responses. Numerous studies emphasized the immunostimulatory properties through the cGAS-STING pathway (109).

##### 3.2.2.5 TLS formation

Gut microbiota-mediated regulation in immune cells may also be through TLSs. TLSs are important tissues to generate adaptive T and B cells. Both the gut microbiota and its metabolites can potentially promote mature TLS formation (110). Signatures of mature TLSs have been described in other papers (111, 112). They have typical structures, which are comprised of an internal B-cell zone surrounded by a T-cell-rich area, which includes CD8 cytotoxic T, CD4 Th-1, and Tfh lymphocytes, as well as LAMP3^+^ DCs (113). However, the composition and distribution of TLSs is different in various tumor types (114) from disorganized cellular aggregates such as early or immature TLSs to mature TLSs (111, 115). Lymphoid aggregates only consisted of a few B cells and T cells without follicular dendritic cells (FDCs) (112). In immature TLS (iTLS), there often are aggregates of immune-suppressive cells such as Treg cells (116, 117), rDCs (116), rMACs (116), PD-1^high^ CD8 T cells (116, 118), and CD4 T cells (118), which can suppress antitumor immunity.

### 3.3 Regulation of the immune system on tumors

The immune system of individuals influences tumorigenesis. Tumor immune responses, including antitumor immunity and tumor immune tolerance, have been widely reported (119). The role of the immune system in eradicating cancer cells has also been reviewed (120, 121). From preclinical to clinical research, evidence has established that the gut microbiota can modulate immunity against tumors and influence the efficacy of cancer immunotherapies, especially immune checkpoint inhibitors (63, 98, 122).

Taken together, the nervous system–gut microbiota–immune system axis should be a good target for preventing tumors.

## 4 Direct regulation of the gut microbiota on tumors

**Figure 4:**
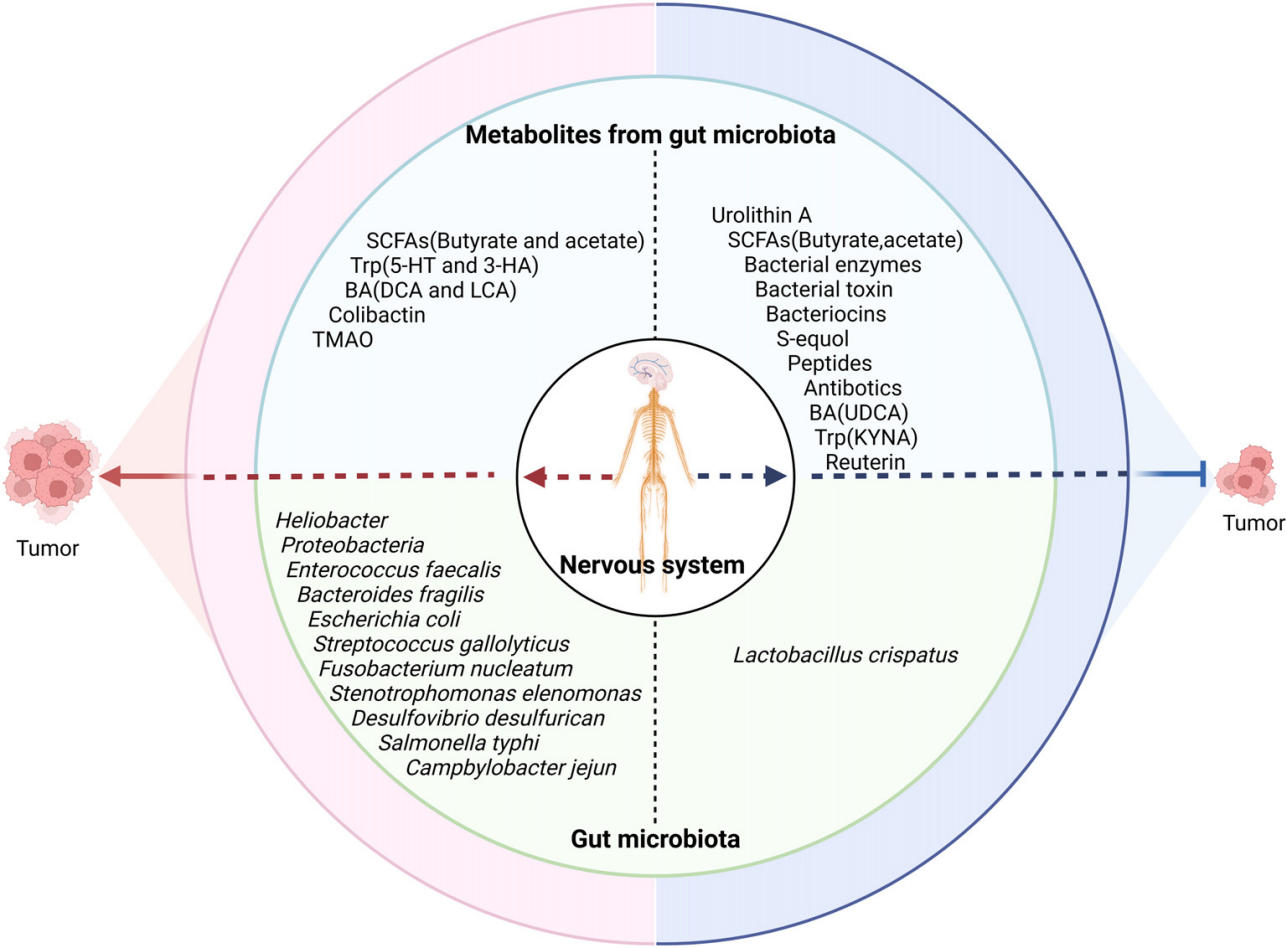
Effects of the nervous system on the tumor through the gut microbiota. The nervous system influences tumor development through gut microbiota/metabolites. SCFAs, short chain fatty acids; Trp, tryptophan; BA, bile acid; DCA, deoxycholic acid; LCA, lithocholic acid; TMAO, trimethylamine N-oxide; UDCA, ursodeoxycholic acid; KYNA, kynurenic acid.

Studies have found that gut microbiota or its metabolites can directly affect tumorigenesis and tumor development (**Figure 4**) (63). Indeed, *Heliobacter pylori* (*H. pylori*) made a well-established contribution in carcinogenesis of gastric cancer. Specific bacteria associated with the onset and progression of colorectal carcinoma (CRC) have also been found, such as *F. nucleatum*, *E. faecalis*, *E. coli*, *S. gallolyticus*, and *B. fragilis*. They used different mechanisms to cause cancers, e.g., *E. coli* (123), enterotoxigenic *B. fragilis* (124), *F. nucleatum* (125), and *Stenotrophomonas elenomonas* (126) promoted tumor development through inflammatory responses, whereas colibactin-expressing *E. coli* (127), cytolethal toxin-expressing *Proteobacteria* (128), and EspF-expressing *E. coli* (129, 130) could cause damage of genetic substances in the cells to cause tumors.

Gut microbiota-derived metabolites can also contribute to tumorigenesis and tumor development such as SCFAs (131), the BA derivatives LCA and DCA (132), and the tryptophan metabolites 5-HT and 3-HAA (133). In addition, the metabolites trimethylamine n-oxide (TMAO) (134) and colibactin from gut commensal *pks+ E. coli* (135) were also related to tumorigenesis. Colibactin, a mutagenic compound by *E. coli*, induced DNA double-strand breaks to promote colorectal cancer development (127). Peptidyl aldehydes from *E. coli* and *Bacillus subtilis* could enhance carcinogenicity by inhibiting protease activity (136).

However, gut microbiota is also negatively related to tumor development, e.g., the abundance of *Lactobacillus crispatus* was negatively correlated with the development of ovarian and breast cancers (131). Gut microbiota metabolites such as the secondary BA UDCA, kynurenic acid (137), *L. reuteri*-derived reuterin (138), purine metabolites from *A. muciniphila* and *B. pseudolongum* (139), and urolithin A (UA) (140) could also inhibit tumor development. Gut microbiota-derived bacteriocins, bacterial toxins, peptides, antibiotics, and bacterial enzymes also have direct effects on cancer. The metabolites from thiopeptides by C*lostridia* and *Lactobacillus* also displayed anticancer effects through proteasome targeting (141).

## 5 Regulation of the nervous system-mediated immune system on tumors

**Figure 5:**
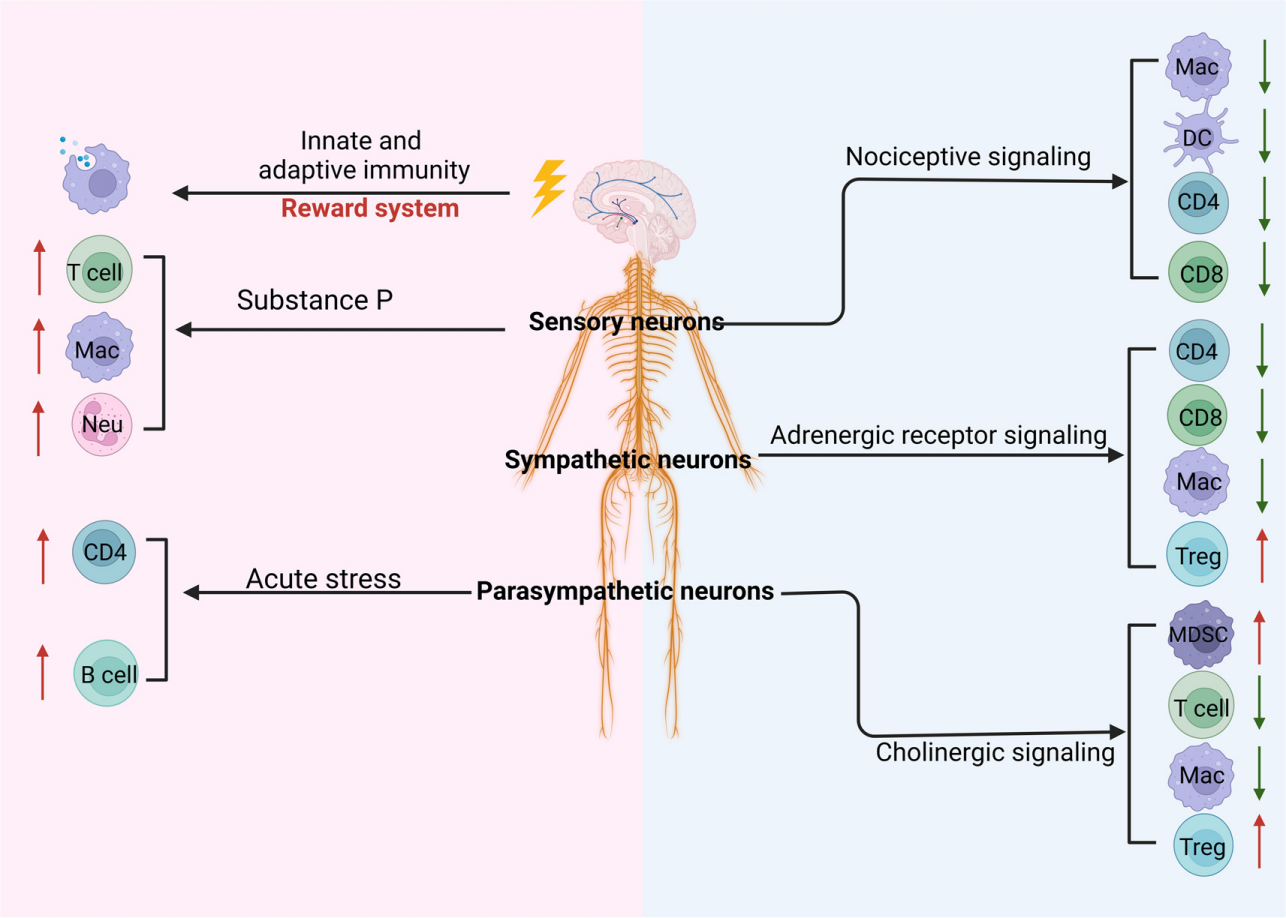
Regulation of the CNS and PNS on the tumor through immune cells. Stimulating the reward system in the central nervous system (CNS) induces innate and adaptive immunity. The peripheral nervous system such as parasympathetic neurons, sympathetic neurons, and sensory neurons can regulate the differentiation and development of immune cells through cholinergic, adrenergic, and nociceptive signaling, respectively. These signaling pathways can inhibit immunity against the tumor through downregulating innate and adaptive immune cells such as macrophages (MACs), dendritic cells (DCs), and CD4 and upregulating immune regulatory cells such as regulatory T cells (Tregs) and myeloid-derived suppressor cells (MDSCs). In addition, sensory neuron-derived substance P can promote the differentiation and function of T cells, MACs, and neutrophils (Neu). Notably, acute stress can also promote CD4 and B-cell immune responses.

Interactions among cancer cells, nerves, and immune cells can regulate overall tumor progression (**Figure 5**) (142).

### 5.1 Peripheral nervous system

In tumors, all types of peripheral nerves, namely, sensory, parasympathetic, and sympathetic nerves, have been detected (30, 31). Clinical studies have indicated the potential of targeting the peripheral nervous system for promoting antitumor immune responses (143).

#### 5.1.1 Parasympathetic nervous system

The parasympathetic/vagal nerve is not only involved in the production of inflammatory factors but also in the regulation of the immune system (37). This is mainly achieved through binding of the neurotransmitter acetylcholine to nicotinic-type receptors or muscarinic-type receptors (144). The vagus nerve-derived acetylcholine exerted its effect through the α7 nicotinic acetylcholine receptor (α7nAChR), which was found on macrophages, inhibiting the production and release of pro-inflammatory cytokines like TNF, IL-1β, IL-6, and IL-18 (145, 146). Muscarinic Ach receptors (mAchRs) were also involved in cholinergic regulation of the immune system (147). It could lead to activation of Treg cells and their infiltration into tissues in the periphery (147). The administration of bethanechol, a mAchR agonist, caused suppression of tumor growth via the mAchR-dependent signaling pathway. Cholinergic vagal signaling also suppressed inflammation to promote colon cancer development through the release of trefoil factor 2 from splenic memory T cells (148). Cholinergic nerve deprivation by vagotomy, the surgical removal of the vagus nerve, facilitated the development of gastric and pancreatic tumors, suggesting that cholinergic signals suppressed tumorigenesis (29).

#### 5.1.2 Sympathetic nervous system

Norepinephrine released locally from sympathetic nerves or systemically from the adrenal gland preferentially binds to α-adrenergic receptors (ADRs), such as the α2-ADR (ADRA2) but also the β1-ADR (ADRB1), β2-ADR (ADRB2), and β3-ADR (ADRB3) (149). It could bind to β2-AR on immune cells to exert immune-suppressive effects, including upregulating PD-1 expression (150) and regulating the function of MDSCs and macrophages (26, 151) to limit antitumor immunity (152) and promote T-lymphocyte exhaustion (153). While β2-AR signaling was disrupted, CD8^+^ T cells exhibited enhanced cytotoxic effector function and migratory capacity (154). During metabolic reprogramming of T cells, β2-AR deficiency also increased mitochondrial membrane potential and biogenesis (155). Through sympathetic innervation, β2-AR signaling also regulated the trafficking of lymphocytes in lymph nodes (156) and egress of hematopoietic stem cells from the bone marrow into the circulation (157), as well as movement of immune cells within tissues (152). The norepinephrine from sympathetic nerves was shown to inhibit NK cell activity via the β-adrenergic receptor (158). Notably, acute stress could trigger adrenergic receptors on CD4^+^ T lymphocytes and B cells to enhance the production of antibody IgG1 and interferons (IFNs).

#### 5.1.3 Sensory neurons

Sensory neurons can activate or suppress host defense and immunity against pathogens, depending on the tissue and disease state. Nociceptive signaling such as the release of calcitonin gene-related peptide (CGRP) and substance P from peripheral nerve fibers could regulate immune responses to affect the development of tumors (159, 160). Substance P (SP), a sensory neurotransmitter, could enhance inflammatory reactions by stimulating the synthesis of pro-inflammatory cytokines (161). SP signaling can promote hematopoietic stem cell activity and hematopoiesis (162) and the survival of active T lymphocytes. *In-vivo* stimulation with SP has been associated with an increased antitumor immune response via an enhanced function of NK cells (163). CGRP downregulated inflammatory cytokine in macrophages and dendritic cells (DCs) and reduced antigen presentation to effector T cells (164). It also led to the release of anti-inflammatory IL-10 from macrophages and decreased effector CD4^+^ and CD8^+^ T lymphocytes via the receptor Ramp1 signaling pathway. Using CGRP-knockout (CGRPKO) mice, reduced tumors were observed in syngeneic oral cancer models (165). The tumor tissue from CGRP^KO^ mice also had a significant increase in tumor-infiltrating CD4^+^ T cells, cytotoxic CD8^+^ T cells, and NK1.1^+^ NK cells as compared to wild-type mice (165). In addition to SP and CGRP, other neurotransmitters such as gamma-aminobutyric acid (GABA), commonly known as serotonin, also play a crucial role in regulating tumor immunity. Studies have shown that supplementing with the neurotransmitter GABA can decrease cAMP levels and inhibit the release of the pro-inflammatory cytokine IL-6.

Notably, the crosstalk between the ENS and immune cells such as macrophages, T cells, and innate lymphoid cells (ILCs) exerts critical roles in maintaining homeostasis.

### 5.2 Central nervous system

The CNS harbors its own special immune system, which is composed of microglia in the parenchyma, MACs, DCs, monocytes, and the barrier systems within the brain (166). However, the CNS also encodes specific immune responses (167). Activation of the reward system can boost innate and adaptive immunity (168–171). The activation of the ventral tegmental area (VTA), a key component of the reward system, could strengthen the host’s immunological defense (168). The optogenetic stimulation of dopaminergic projections from the VTA to the medial prefrontal cortex could attenuate stress-mediated aversive effects on tumor growth in a breast cancer model (172). The repetitive optogenetic activation of the VTA tyrosine hydroxylase (TH) in the medial prefrontal cortex attenuated stress-induced progression of breast cancers and reduced serum concentration of norepinephrine and corticosterone. In models of melanoma and lung cancer, VTA activation could reduce tumor growth by altering the functional profile of MDSCs (169). The periventricular hypothalamic corticotropin hormone neurons could regulate the trafficking of monocytes and lymphocytes between peripheral tissues and bone marrow by activating the hypothalamic–pituitary–adrenal axis, and they influence the adaptive immune responses by ultimately projecting to the splenic nerve. Central neurotransmitters such as GABA also reduced immunity against tumors (173, 174). In a mouse model of colon cancer, GABA could bind to GABA_A_ receptors on CD8^+^ T lymphocytes to reduce antitumor immunity and enable tumor growth (173).

Notably, Schwann cells (SCs) in the CNS, specifically nerve repair subtypes, can enhance immune cell chemotaxis by the release of chemokines (175), e.g., SCs can promote the recruitment and immuno-inhibitory function of MDSCs and release C-C motif chemokine ligand 2 (CCL2) to aid in cancer progression. Although cultured with a melanoma-conditioned medium, glial fibrillary acidic protein^+^ (GFAP^+^) SCs could also promote mRNA levels of genes, involved in immune surveillance and chemotaxis such as IL-6, transforming growth factor (TGF)-β, and vascular endothelial growth factor (VEGF).

## 6 Therapeutic potential of the nervous system–gut microbiota–immune system axis on tumors

Tumor-innervating peripheral nerve fibers have recently been found to be associated with tumor development (176). The therapeutic means based on these findings have been developed, e.g., pharmacological blocking of β-adrenergic signaling was shown to enhance CD8^+^ T-cell infiltration into the TME and to improve the response to immune checkpoint blockade (177). The use of non-selective β1-blockers and β2-blockers has shown effectiveness in improving treatment outcomes across various human cancer types such as melanoma, prostate, breast, pancreatic, ovarian, non-small-cell lung cancer, and colorectal cancer (176).

Since gut microbiome alterations are associated with various cancers and the gut microbiota can communicate with the brain via a bidirectional pathway (178), the nervous system–gut microbiota–immune system axis should also become a target for developing new therapeutic method(s) for preventing tumors. With the advancement in the nervous system–gut microbiota–immune system axis, it is possible to affect nervous signals or nervous transmitters to treat tumors, e.g., the vagus nerve, involved in stress response, can affect the composition of the gut microbiota. Targeting these properties of the vagus nerve could be of interest in preventing tumors. Thus, changing the composition of the gut microbiota through modulating nervous system-associated factors such as neurotransmitters and neuropeptides could potentially improve tumor therapy.

Importantly, specific gut microorganism(s) controlled by the nervous system is identified, and the interventions targeting the gut microbiota, including fecal microbiota transplantation, probiotics, prebiotics, engineered bacteria, and dietary interventions, can be used to treat tumors. The gut microbiome has become an efficient target for making cancer chemotherapy/immunotherapy safer or efficient, which will further improve cancer survival rates in the future.

In addition, specific gut microorganism(s) can regulate the differentiation and function of immune cells through the receptor signal, epigenetic modification, metabolism reprogramming, cGAS-STING pathway, and TLS formation. The inhibitor or promoter, which affects these processes, can also be a potential mean(s) to control tumorigenesis and tumor development.

## 7 Conclusion and perspective

While existing studies have highlighted the involvement of both sympathetic and parasympathetic nerves in pro- and antitumor immunity, here, we have explored the potential effects of the nervous system–gut microbiota–immune system axis on tumorigenesis and tumor development. The nervous system regulates immune cells through the gut microbiota, which can prevent tumor development, including the effects of the nervous system on gut microbiota, gut microbiota on the immune system, and the immune system on tumors. The ENS is essential for the regulation of gut microbiota. There exists also altered gut microbiota in anxiety, depression, and stress. Gut microbiota has wide effects on the function and differentiation of immune-effective cells such as effective CD8 and NK cells, immune regulatory (suppressive) cells such as Tregs, and also inflammatory cells such as Th1, Th2, and Th17. These gut microbiota-associated immune cells can deeply affect the occurrence and development of tumors.

The regulation in the gut microbiota by the nervous system can potentially become a target for preventing tumor. Thus, the identification of changes in the gut microbiome associated with the nervous system may provide valuable information in the choice of treatment. However, the characteristics, function, and composition of the gut microbiota, which are controlled by the nervous system, remains to be further understood. With the emergence of gut microbiota as an important driver of disease, there is now a unique opportunity to target microbiota in the gut to achieve precision in cancer care (179, 180).

In conclusion, a better understanding of the role of the central and peripheral nervous system and the influence of innervation on the components of the gut microbiota will strengthen the therapeutic armamentarium against tumors.
